# Supplementary material for: A UMLS-based spell checker for natural language processing in vaccine safety
Source: BMC Med Inform Decis Mak. 2007 Feb 12;7:3. doi: 10.1186/1472-6947-7-3 (PMC1805499; doi:10.1186/1472-6947-7-3)
Supplement: Additional file 13 — RAP application directory. Sets up directory for RAP (RDF application for PHP) source code [file 1472-6947-7-3-S13.gz › rap/doc/phpdoc/__filesource/fsource_model__modelNode.php.html]

xml version="1.0" encoding="iso-8859-1"?


File Source for Node.php


# Source for file Node.php

Documentation is available at Node.php

```
1. <?php

3. // ----------------------------------------------------------------------------------
4. // Class: Node
5. // ----------------------------------------------------------------------------------


9. /**
10. * An abstract RDF node.
11. * Can either be resource, literal or blank node.
12. * Node is used in some comparisons like is_a($obj, "Node"),
13. * meaning is $obj a resource, blank node or literal.
14. *
15. * <BR><BR>History:<UL>
16. * <li>09-10-2002                : First version of this class.</li>
17. * </UL>
18. *
19. * @version  V0.9.1
20. * @author Chris Bizer <chris@bizer.de>
21. *
22. * @todo nothing
23. * @package model
24. * @abstract
25. *
26. */
27. class Node extends Object {
28. } // end:RDFNode


31. ?>
```

Documentation generated on Thu, 7 Jul 2005 13:42:17 +0200 by phpDocumentor 1.3.0RC3
